# Elixhauser Comorbidity Index to Predict Perioperative Bleeding and Adverse Spine Surgery Outcomes

**DOI:** 10.3390/jcm15051791

**Published:** 2026-02-27

**Authors:** Mitchell K. Ng, Michael A. Mont, Mosadoluwa Afolabi, Prathiksha N. V, Amitha Kumar, Stephen S. Johnston

**Affiliations:** 1 Rothman Orthopaedic Institute, Thomas Jefferson University Hospital, Philadelphia, PA 19107, USA; 2 Department of Orthopaedic Surgery, The Rubin Institute for Advanced Orthopedics, Baltimore, MD 21215, USA; rhondamont@aol.com; 3 Global Epidemiology Organization, MedTech Surgery, Johnson & Johnson, New Brunswick, NJ 08933, USA; 4 Mu Sigma, Whitefield Road, Bangalore 560066, Karnataka, India; 5 Health Economics and Market Access, MedTech Surgery, Johnson & Johnson, Raritan, NJ 08869, USA

**Keywords:** spine surgery, disruptive bleeding, the elixhauser comorbidity index, mortality, ventilator use, lengths of stay, readmissions, hospital costs

## Abstract

**Introduction:** As spine surgery volume continues to grow, ensuring patient safety and minimizing complications are increasingly critical. Disruptive bleeding—defined as hemorrhagic events requiring clinical intervention—is a significant perioperative challenge. This study aimed to: (1) quantify disruptive bleeding incidence; (2) evaluate associations between patient demographics, Elixhauser Comorbidity Index (ECI), and bleeding risk; and (3) assess the impact of disruptive bleeding on mortality, ventilator use, length of inpatient stay, 90-day readmissions, and inpatient costs. **Methods:** A nationwide healthcare database was used to identify patients who underwent spine surgery in 2019. Patients were subdivided by the Elixhauser Comorbidity Index (ECI) from 0 to ≥6, and multivariate logistic regression was employed to analyze for potential association with disruptive bleeding. Odds ratios (ORs) and corresponding 95% confidence intervals (CIs) were calculated for each ECI classification. After controlling for baseline demographics, generalized linear models were used to evaluate how disruptive bleeding influenced hospital mortality, ventilator use, 90-day readmission rates, lengths of inpatient stay, and inpatient costs. **Results:** Among 165,461 patients undergoing spine surgery, 15,337 (9.3%) experienced disruptive bleeding. Women and Medicare coverage were associated with higher bleeding risk (*p* < 0.05). Disruptive bleeding odds increased with comorbidity burden, ranging from OR = 2.31 (95% CI 1.92–2.77) for ECI = 5 to OR = 3.32 (95% CI 2.73–4.06) for ECI ≥ 6. Disruptive bleeding was associated with increased ventilator use (18.4 versus 8.2% for ECI ≥ 6; *p* < 0.001) and inpatient mortality (3.0 versus 0.7% for ECI ≥ 6; *p* < 0.001). Hospital stays were significantly prolonged (10.4 versus 6.6 days for ECI ≥ 6; *p* < 0.001), 90-day readmission rates were higher (19.8 versus 14.7%; *p* < 0.001), and inpatient costs increased substantially ($68,000 versus $37,500; *p* < 0.001). **Conclusions:** Disruptive bleeding in spine surgery is more frequent among patients with elevated comorbidity burdens and is linked to greater mortality, ventilator dependence, and healthcare resource use. These findings highlight the importance of proactive risk stratification and targeted perioperative management strategies for high-risk patients undergoing spine surgery.

## 1. Introduction

Spine surgery represents a high-volume subspecialty in orthopaedic surgery and neurosurgery, with increasing volumes driven by an aging population, rising rates of degenerative spinal pathology, and growing surgical indications [1,2]. Over the past decade, volume of elective lumbar fusion has increased 62.3% (32.1 per 100,000 U.S. adults), with a 138.7% increase in surgical volume amongst patients aged 65 or older [3]. Common spine procedures such as discectomy, laminectomy, and spinal fusion are widely utilized to address conditions ranging from herniated discs and spinal stenosis to deformity and instability [1,4]. While these procedures can lead to significant improvements in pain and function, they are also associated with considerable perioperative risk [5]. One of the most concerning complications is perioperative bleeding, particularly in the context of spine surgery where extensive dissection, proximity to vascular structures, and instrumentation can result in blood loss [5]. Reported intraoperative blood loss can vary widely depending on procedure type and patient factors, and the need for transfusion remains common in complex or multilevel cases. For a primary lumbar decompression and spinal fusion mean total blood loss is estimated to average 300–800 mL for routine 1–2 level procedures, with higher volumes (range 360–3100 mL) in multi-level or more extensive posterior cases [6,7,8,9,10].

Of particular clinical importance is disruptive bleeding, a subset of major bleeding events that can lead to worsened clinical outcomes. Disruptive bleeding is identifiable through diagnosis codes indicating hemorrhage or hematoma as surgical complications, procedural codes for hemostatic interventions, and billing records for blood product use or drain placement [11]. Although some perioperative bleeding is expected, disruptive bleeding has been linked to higher rates of infection, thromboembolic complications, respiratory failure requiring ventilator support, extended hospitalizations, and increased healthcare costs [5,7,9]. A meta-analysis of 8 studies including 34,185 patients undergoing spine surgery found perioperative blood transfusion was a risk factor for postoperative infection (Odds Ratio (OR): 2.99; 95% CI, 1.95 to 4.59; *I*^2^ = 86%) [12]. Despite its relevance, limited data exist on the incidence and impact of disruptive bleeding specifically within the context of spine surgery. Moreover, patients with a greater burden of comorbid disease may be especially vulnerable to bleeding complications due to impaired physiological reserve, anticoagulant use, or delayed wound healing [13].

Efforts to minimize perioperative blood loss in spine surgery typically involve a multimodal approach spanning the preoperative, intraoperative, and postoperative phases of care [5,14]. Preoperatively, optimization includes discontinuation or bridging of anticoagulant and antiplatelet agents, correction of anemia or coagulopathy, and careful blood pressure control. Preoperative screening for bleeding disorders and comorbidity optimization, particularly for conditions like renal insufficiency or liver disease, can further reduce risk [15,16]. Intraoperative strategies focus on minimizing tissue trauma, utilizing meticulous dissection techniques, maintaining normothermia, and applying antifibrinolytic agents such as tranexamic acid (TXA), which has been shown to reduce intraoperative bleeding and transfusion requirements [17]. Use of cell salvage, hypotensive anesthesia during subperiosteal dissection, electrocautery, and hemostatic agents (e.g., fibrin sealants or oxidized cellulose) also supports intraoperative hemostasis. Postoperatively, management includes continued monitoring for hematoma formation, judicious use of surgical drains, repeat TXA dosing when appropriate, and cautious timing of thromboprophylaxis initiation [13,14,17,18]. A substantial proportion of patients, particularly those with complex comorbidities, nevertheless remain at risk for disruptive bleeding, highlighting the need for improved risk stratification and targeted interventions.

This study aims to address these gaps by leveraging real-world data from a large national database to evaluate the burden of disruptive bleeding in spine surgery. The primary objectives are to: (1) determine the incidence of disruptive bleeding across a large cohort of patients undergoing spinal procedures; (2) assess the association between comorbidity burden (measured by the Elixhauser Index and bleeding risk); and (3) quantify the downstream effects of disruptive bleeding on outcomes including mortality, ventilator use, lengths of stay, 90-day readmission, and total hospital costs. We hypothesized that increasing Elixhauser comorbidity burden is associated with a graded increase in disruptive bleeding risk and subsequent adverse clinical and economic outcomes.

## 2. Methods

### 2.1. Premier Database

This study was based on a retrospective observational design, utilizing real-world discharge-level data obtained from the Premier Healthcare Database (PHD) [19,20]. The PHD compiles billing records from inpatient and outpatient encounters across over 1000 U.S. hospitals/healthcare systems that were affiliated with the Premier Healthcare Performance Improvement Alliance [21,22,23,24]. Although federally funded hospitals are not represented in the dataset, the PHD is widely recognized as nationally representative due to its inclusion of diverse hospital types—varying in size, geographic location, rural or urban status, and academic affiliation. The database contains a wide range of clinical and administrative information, such as patient demographics, primary and secondary diagnoses, details of medical interventions (including procedures, pharmaceuticals, and devices), lengths of stay, and discharge status. It also includes attributes related to the institutions and healthcare providers involved in each encounter. Furthermore, the dataset features cost data for each hospitalization, with detailed, itemized charges recorded by hospital cost-accounting systems.

The analysis of this dataset was conducted under an Institutional Review Board (IRB) exemption in accordance with Title 45 Code of Federal Regulations (45 CFR 46.104(d)(4)(ii)), permitting the use of de-identified data for research purposes [21]. Because the PHD excludes identifiable information pertaining to patients, institutions, and clinicians, formal IRB approval was not required.

### 2.2. Patient Cohort

Patients included in this study were aged 18+ years at the time of undergoing inpatient or outpatient spine surgery in 2019, with the first documented procedure designated as the index event. To allow for sufficient follow-up, only patients whose index surgery occurred at hospitals that continued to contribute data to the Premier Healthcare Database (PHD) for a minimum of 90 days after discharge were considered eligible. The study exclusion criteria included patients transferred from another facility for their index procedure, patients with missing data (discharge status or sex), and cases with zero/negative total charges related to overall costs, room and board, and/or supplies.

### 2.3. Patient Categories, Study Variables, and Outcome Measures

The Elixhauser Comorbidity Index, a validated and widely used method for evaluating coexisting medical conditions in real-world datasets, was employed to assess patient comorbidity burden [19,20]. This index encompasses 31 discrete comorbidities and is commonly applied in health services and outcomes research. Comorbid conditions were identified using ICD-10-CM diagnosis codes flagged as present on admission during the index hospitalization. For analytical stratification, patients were grouped based on the number of comorbidities: 0, 1, 2, 3, 4, 5, or ≥6. This count-based stratification was selected to evaluate cumulative medical complexity and its dose-response association with disruptive bleeding and healthcare utilization outcomes.

A comprehensive set of covariates was included to describe the study population and control for confounding in multivariable analyses. These included demographic variables (age, sex, race, ethnicity), primary payer type, and/or institutional characteristics such as hospital size (bed count), teaching status, geographic region, and urban versus rural location. Additional operational and provider-level factors—such as hospital cost structure, care setting, surgeon location, and procedural volume for the index surgery—were also considered.

Disruptive bleeding is defined as perioperative hemorrhage adversely affecting clinical outcomes or increased healthcare utilization. These events were identified during the index hospitalization through several indicators, including:

- ICD-10-CM diagnosis codes indicating hemorrhage/hematoma as a surgical complication,
- ICD-10-PCS procedure codes for intraoperative or postoperative hemostatic interventions,
- Hospital billing records for Hemovac drain utilization,
- Charges related to blood transfusion and erythropoietin administration, and
- Costs associated with blood products such as red blood cells, cryoprecipitates, platelets, fresh frozen plasma, and whole blood.

Furthermore, transfusion-related complications, such as infections, acute kidney injury, or thromboembolic events, were captured using relevant ICD-10-CM codes during the initial hospital stay [22]. Additional outcomes assessed included total hospital costs (reflecting institutional expenses), lengths of stay for the index admission, and 90-day all-cause inpatient readmission rates.

Overall, a total of 165,641 patients aged 18+ years who underwent spine surgery between 1 January 2019 and 31 December 2019 were identified (Table 1). The majority underwent spine fusion (67.9%), followed by spine laminectomy (27.1%), and spine discectomy (4.9%). The mean age of the study cohort was 59 years, consisted primarily of male patients (50.8%), White (81.7%), non-Hispanic ethnicity (77.9%), followed by African American (8.8%), married (57.0%), and covered by Medicare (43.9%). Regarding surgical settings, most of the spine procedures (59.4%) were performed in the inpatient setting, in a hospital with 500+ beds (36.4%), teaching hospital (52.5%), located in an urban setting (92.0%), with 500+ provider volume (35.0%), and provider region predominantly in the south (35.0%). The majority of study patients had an Elixhauser score between 0 and 2, while 3% had a score of ≥6 (0 = 22.3%, 1 = 24.8%, 2 = 22.6%, 3 = 15.3%, 4 = 8.1%, 5 = 3.9%, ≥6 = 2.9%). The distribution of patients by Elixhauser score differed between study cohorts.

### 2.4. Data Analyses

Categorical variables were summarized using patient counts and corresponding percentages within each group, while continuous variables were reported as means with standard deviations for normally distributed data or as medians with additional distributional statistics for non-normally distributed data. Generalized linear models (GLMs) were used to examine associations between comorbidity burdens and clinical outcomes, with appropriate covariate adjustments including demographic, payer, and hospital characteristics to decrease potential confounding. Separate GLMs employing logit links and binomial distributions estimated the impact of comorbidity burdens on the likelihood of experiencing disruptive bleeding and transfusion-related complications. To assess whether comorbidity burdens modified the effects of disruptive bleeding on total hospital costs, index hospital lengths of stay, and 90-day readmission rates, additional GLMs were constructed with interaction terms between comorbidity burdens and disruptive bleeding. Model selection was based on the distribution of each outcome variable, using log links with gamma distributions for cost analyses, log links with negative binomial distributions for lengths of stay, and logit links with binomial distributions for readmission outcomes. Analyses of hospital lengths of stay were limited to inpatient procedures (*n* = 106,963). All statistical tests were two-tailed, with significance defined as *p* ≤ 0.05. Data analysis was performed using Statistics and Data (Stata) SE 16 (StataCorp, College Station, TX, USA).

## 3. Results

### 3.1. Disruptive Bleeding Incidence and Associated Demographics

Among 165,461 patients undergoing spine surgery, 15,337 (9.3%) experienced disruptive bleeding. Patient demographics were dissimilar for the disruptive bleeding group when compared to the non-disruptive group for age (62 versus 59 years), race (11.4 versus 8.5% African-American), and insurance group (52.7 versus 43.1% Medicare).

### 3.2. Higher Elixhauser Comorbidity Score Associated with Increased Risk of Disruptive Bleeding

Patients with disruptive bleeding had a higher comorbidity burden overall (Table 2). When considering patients with disruptive bleeding, 40% had an Elixhauser score of 3+, compared to only 27% in the non-disruptive bleeding group.

**Figure 1:**
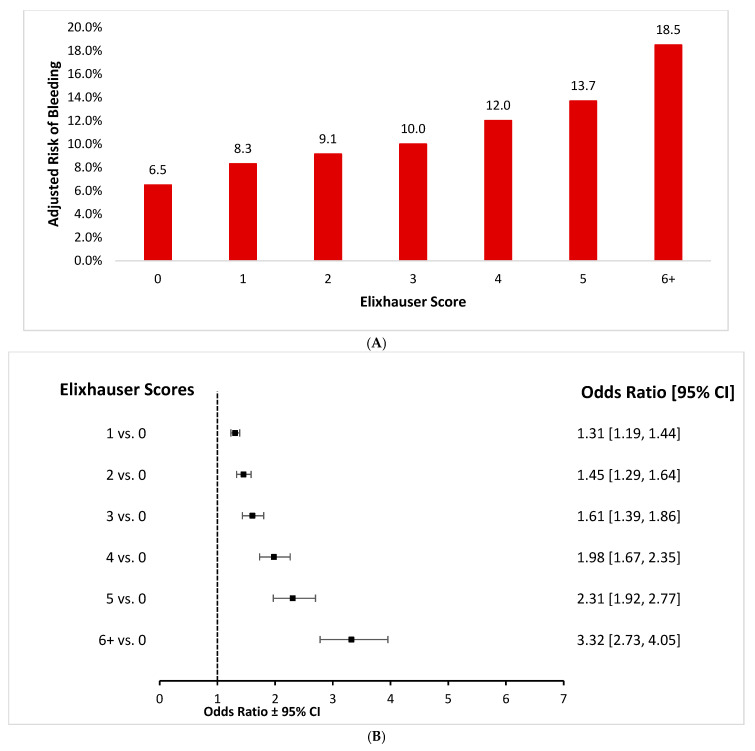
Risk of bleeding divided by Elixhauser score. (**A**) Absolute risks. (**B**) Odds ratios.

Multivariate-adjusted odds of disruptive bleeding increased with an increasing number of comorbidities (Figure 1A,B). Patients with an Elixhauser score of 5 had 2.3X higher odds of disruptive bleeding relative to those who had a score of 0 (odds ratio [OR] = 2.31, 95% confidence interval [95% CI] = 1.92–2.77). Patients with an Elixhauser score of 6 had 3.3X odds of disruptive bleeding relative to patients who scored 0 (odds ratio [OR] = 3.32, 95% confidence interval [95% CI] = 2.73–4.06).

**Figure 2:**
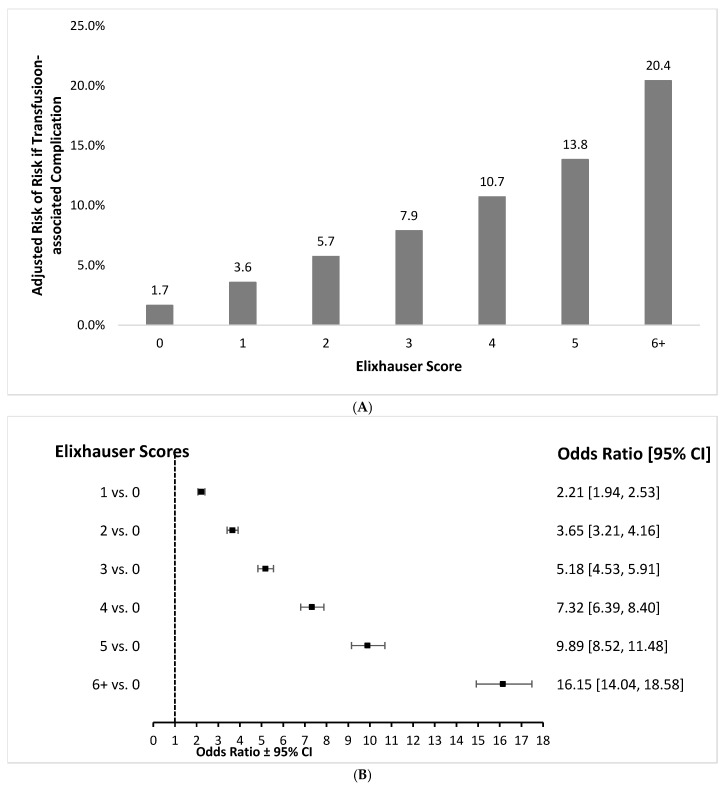
Risk of transfusion-associated complication events by the Elixhauser score. (**A**) Absolute risks and (**B**) Odds ratios.

Similarly, risk of transfusion-associated complications significantly increased as the number of comorbidities increased (Figure 2A,B). Odds of a transfusion-associated complication were approximately tenfold higher in patients who had an Elixhauser score of 5 versus those who scored 0 (OR = 9.89, 95% CI = 8.52–11.48). Overall transfusion-associated complication rates among patients who had scores of at least 6 were more than 16-fold that of patients who had scores of 0 (OR = 16.15, 95% CI = 14.04–18.56).

### 3.3. Disruptive Bleeding Associated with Increased Risk of Death

**Figure 3:**
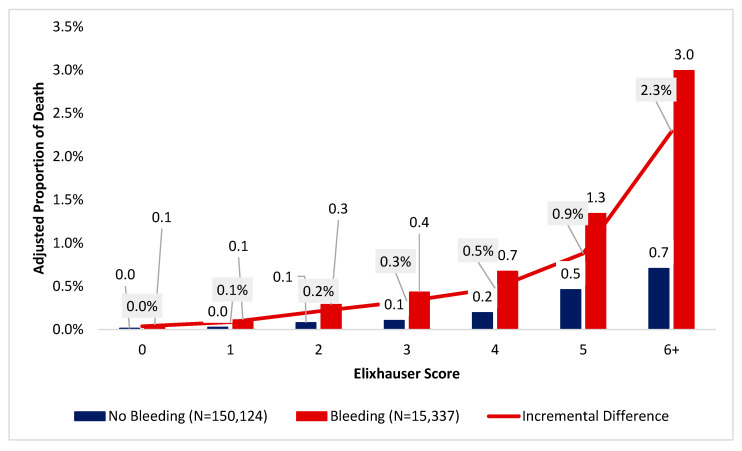
Incremental differences in proportion of death among patients undergoing inpatient procedures, divided by Elixhauser score or occurrence of disruptive bleeding.

In addition, patients with disruptive bleeding had increased odds of death relative to those in the non-disruptive bleeding cohort, who had a higher proportion of deaths (Figure 3). The greatest incremental difference between patients who have versus those who do not have disruptive bleeding was highest among patients whose Elixhauser score was 6 (3.0% [95% CI = 1.9–4.1%] versus 0.7% [95% CI = 0.4–1.0%]).

### 3.4. Disruptive Bleeding Associated with Increased Lengths of Stay

**Figure 4:**
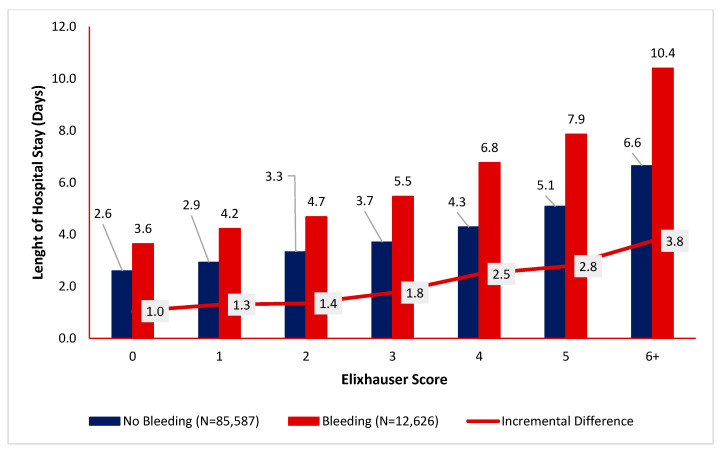
Differences in index encounter lengths of stay among patients undergoing inpatient procedures, by the Elixhauser score, and occurrence of disruptive bleeding.

Among patients undergoing an inpatient procedure, the occurrence of disruptive bleeding also had a noticeable impact on the index encounter’s adjusted length of stay (Figure 4). At every comorbidity level, patients with disruptive bleeding had longer stays than those without disruptive bleeding. The greatest incremental difference in lengths of stay between patients who had versus did not have disruptive bleeding was in patients with Elixhauser scores of at least 6 (10.4 days [95% CI 6.3–7.0 days] versus 6.6 days [9.6–11.2 days]).

### 3.5. Disruptive Bleeding Associated with Increased Ventilator Use

**Figure 5:**
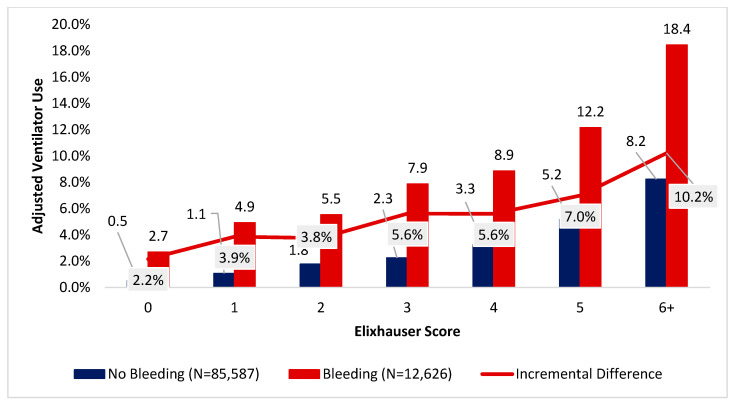
Index encounter differences in inpatient ventilator use among patients undergoing a procedure in the inpatient setting, by Elixhauser score and occurrence of disruptive bleeding.

Ventilator use was higher for patients from the disruptive bleeding cohort relative to those in the non-disruptive bleeding cohort (Figure 5). The greatest noticeable difference between patients who had versus did not have disruptive bleeding was highest among patients with Elixhauser scores of 6 (18.4% [95% CI = 15.4–21.4%] versus 8.2% [95% CI = 7.0–9.4%]).

### 3.6. Presence of Disruptive Bleeding Associated with Subsequent Readmission Risk

**Figure 6:**
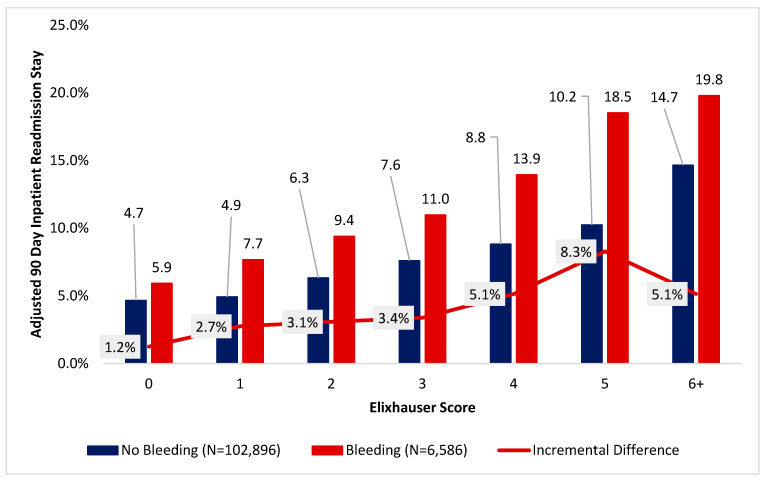
Difference in 90-day inpatient readmission stay among patients undergoing inpatient procedure, sorted by Elixhauser score and occurrence of disruptive bleeding.

The 90-day inpatient readmission rate was greater for patients in the disruptive bleeding cohort relative to those in the non-disruptive bleeding cohort. At every increased Elixhauser score, patients with disruptive bleeding had a higher proportion of 90-day inpatient readmission (Figure 4). Among patients with an Elixhauser score of specifically 5, those who had experienced disruptive bleeding had a 81% higher odds of 90-day inpatient readmission (18.5% versus 10.2%, *p* < 0.05). Among patients with an Elixhauser score of 6, those who had experienced disruptive bleeding had a 35% higher odds of 90-day inpatient readmission (19.8 versus 14.7%, *p* < 0.05) (Figure 6).

### 3.7. Disruptive Bleeding Associated with Higher Hospital Costs

**Figure 7:**
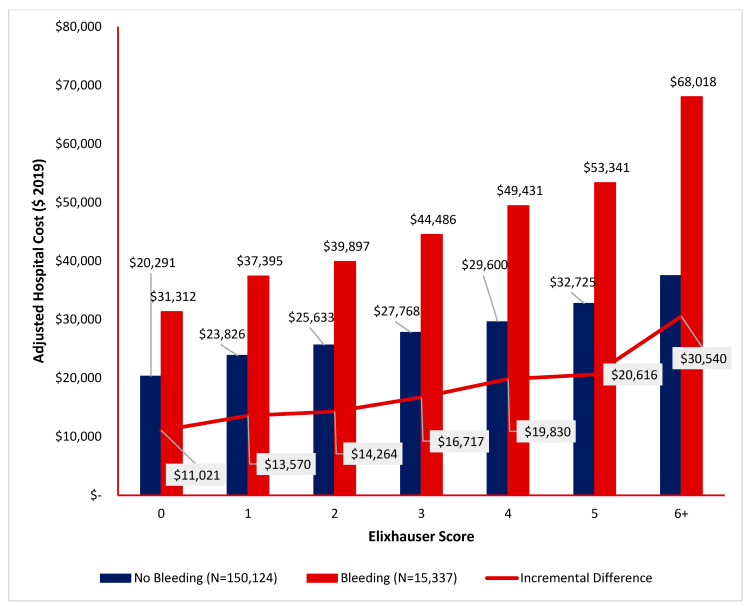
Differences in index encounter total hospital costs, sorted by the Elixhauser score and occurrence of disruptive bleeding.

Increased hospital cost of bleeding was associated with an increased number of comorbidities (Figure 7). At greater Elixhauser scores, patients who had disruptive bleeding had higher mean hospital costs. Among patients with an Elixhauser score of 3, those who had experienced disruptive bleeding during their index encounter incurred higher costs than those who did not have disruptive bleeding: $44,500 [95% CI = $40,200–$48,800] versus $27,800 [95% CI = $26,500–$29,100]. The greatest difference in hospital cost between patients with versus without disruptive bleeding was highest among patients who had Elixhauser scores of at least 6 ($68,000 [95% CI = $62,500–$73,500] versus $37,500 [95% CI = $35,700–$39,300]).

## 4. Discussion

This study aimed to quantify the disruptive bleeding incidence following spine surgery, examine associated patient demographics and comorbidity burden as measured by the Elixhauser Comorbidity Index (ECI), and assess its impact on perioperative healthcare utilization, including hospital lengths of stay, 90-day readmission rates, ventilator use, and hospital costs. Among 165,461 patients who underwent spine procedures, 9.3% experienced disruptive bleeding. While demographic differences such as age, race, and sex were observed between cohorts, the most notable predictors included sex and Medicare insurance coverage. Comorbidity burden emerged as the strongest independent risk factor for disruptive bleeding, with odds ratios increasing progressively from 2.31 (ECI of 5) to 3.32 (ECI ≥ 6) compared to those with an ECI of 0. Disruptive bleeding was also strongly associated with increased healthcare utilization, as evidenced by longer inpatient stays, higher rates of mechanical ventilation and readmissions, and significantly greater hospital costs. These effects were most pronounced among patients with higher ECI scores, highlighting the compounding impact of bleeding and medical complexity.

Our study found that women and Medicare status were independently associated with higher rates of disruptive bleeding. These findings align with prior spine literature, which has consistently identified advanced age and sex as risk factors for intraoperative blood loss and transfusion. A retrospective review of 112 patients undergoing posterior lumbar decompression and fusion demonstrated that age (*p* < 0.05) was associated with intraoperative blood loss and predictive of blood transfusion [23]. A meta-analysis of 22 studies involving adult patients undergoing spine surgery found that patients who received blood transfusions were older, women, and had increased comorbidities [24]. The increased prevalence of anticoagulant use, decreased vascular compliance, and lower baseline hemoglobin levels in older adults may potentially explain these associations.

Our results are consistent with these prior studies and reinforce the association between increasing Elixhauser Comorbidity Index and disruptive bleeding risk. The ECI is a robust, validated instrument used to quantify cumulative medical complexity across a wide range of surgical populations [19]. It has demonstrated superior predictive accuracy over the Charlson Comorbidity Index (CCI) for postoperative mortality and adverse events [19], particularly in high-risk specialties such as orthopaedics and neurosurgery [22,25,26]. A retrospective review of 46,700 patients undergoing posterior cervical decompression and fusion revealed ECI had superior predictive ability to CCI in predicting 8 complications (airway complications, hemorrhagic anemia, cardiac arrest, pulmonary embolism, sepsis, septic shock, and urinary tract infection) over CCI [26]. Despite its growing use, few studies have evaluated the relationship between ECI and bleeding complications specifically in the spine surgery setting (Table 3). Our findings help address this gap and underscore the importance of incorporating comorbidity burden into preoperative bleeding risk assessment.

Specific comorbidities captured by the ECI—including chronic kidney disease, coagulopathy, congestive heart failure, and liver disease—are known to impair clot formation and exacerbate bleeding risk [19]. For example, chronic kidney disease is associated with impaired platelet aggregation and frequent use of antiplatelet medications, while liver dysfunction alters the synthesis of clotting factors. A study of 1834 patients undergoing elective lumbar spine surgery for degenerative lumbar disease found that dialysis-dependent patients had substantially higher inpatient mortality rates (1.8 versus 0.1%; *p* < 0.001) and increased need for blood transfusion (18.3 versus 12.5%; *p* < 0.001) overall [27]. Diabetes mellitus, a common comorbidity among patients undergoing spine surgery, may also contribute to delayed hemostasis through endothelial dysfunction and increased fibrinolysis. A meta-analysis of 40 cohort studies with 2.9 million spine surgeries found that diabetes was associated with subsequent blood transfusions (OR 1.39, 95% CI 1.11–1.75, *p* = 0.005) [28]. Taken together, our findings reflect both the association between increased comorbidity burden and subsequent disruptive bleeding.

**Table 3:** List of Studies Identifying Risk Factors for Disruptive Bleeding during Spine Surgery.

Study	Year	Sample Size	Risk Factors Assessed	Key Findings on Excessive Blood Loss
Daher et al. [29]	2024	552	American Society of Anesthesiologists (ASA) class, preoperative albumin, hypertension	ASA class, low preoperative albumin, and baseline hypertension were risk factors for bleeding and other adverse events.
Mohme et al. [21]	2021	996	Body mass index (BMI), epidural spinal cord compression score (ESCC)	Increased BMI and higher ESCC were associated with risk of needing allogeneic blood transfusion.
White et al. [30]	2018	5805	Age, ASA class, cardiac comorbidity, bleeding disorder	Age > 65 years, ASA class > 3, cardiac comorbidity and bleeding disorder were independent risk factors for blood transfusion.
Basques et al. [31]	2015	4223	Age, ASA class, sex, pulmonary disease, preoperative hematocrit < 36.0, 3 or more levels	Age > 70 years, ASA class > 3, women, pulmonary disease, preoperative hematocrit < 36.0, and multi-level fusion > 3 were associated with increased rates of blood transfusion.
Huang et al. [32]	2015	206	BMI, spinal canal narrowing, spine fusion segments > 1	Increased BMI, extreme spinal canal narrowing, and spine fusion segments > 1 were independent risk factors for significant blood loss.
Yoshihara et al. [33]	2014	162,671	Age, sex, race, weight loss anemia, Elixhauser comorbidity (ECI) index, insurance status	Increased age, women, African-American race, weight loss, increased ECI and Medicare insurance status were significant predictors of allogeneic blood transfusion.
Zheng et al. [23]	2002	112	Age, preoperative hemoglobin, number of levels fused	Older age, low preoperative hemoglobin and number of levels fused were associated with subsequent blood transfusion.

While previous literature has characterized general risk factors for bleeding and transfusion in spine surgery, few studies have directly examined the impact of disruptive bleeding on subsequent outcomes across comorbidity strata. A study of 199 patients undergoing lumbar fusion surgery found that intraoperative blood loss > 500 mL was an independent risk factor for postoperative mortality [34]. In our cohort, patients with ECI ≥ 6 who experienced disruptive bleeding had a 3.0% in-hospital mortality rate compared to 0.7% in those without bleeding.

Length of stay (LOS) is a widely recognized metric of surgical recovery, cost, and complication burden. Our results demonstrate that disruptive bleeding significantly prolongs hospitalization following spine surgery across all comorbidity strata. In patients with an ECI ≥ 6, bleeding was associated with a mean LOS of 10.4 days, nearly 4 days longer than their non-bleeding counterparts. The extended hospitalization likely reflects the need for ongoing hemodynamic monitoring, wound care, physical therapy delay, and evaluation of bleeding-associated sequelae. A single-center study of 1118 patients undergoing spine surgery found that the presence of transfusion was associated with 60% longer stay (adjusted incidence rate ratio = 1.60, *p* < 0.001) [35]. These findings are particularly relevant in bundled payment models, where LOS outliers drive financial penalties.

Our analysis also found that disruptive bleeding significantly increased 90-day inpatient readmission rates, with this effect being most pronounced in patients with intermediate-to-high ECI scores. A retrospective study of 160 patients undergoing elective spine surgery found that patients who received a blood transfusion were three times more likely to be re-admitted within 30 days of hospital discharge (no transfusion 5% versus transfusion 16.67%, *p* = 0.01) [36]. In our study, among patients with ECI = 5, disruptive bleeding raised the 90-day readmission rate from 10.2% to 18.5%. Ultimately, the effects of disruptive bleeding on both increased lengths of stay and higher readmission rates contributes to increased healthcare costs [37,38]. In our study, disruptive bleeding was associated with substantial increases in total hospital costs, particularly among patients with elevated comorbidity burdens. For patients with ECI ≥ 6, mean inpatient costs rose from $37,500 to $68,000 in the presence of bleeding—a nearly 81% increase. As healthcare systems move toward value-based care models, our data provides evidence that perioperative bleeding represents a high-impact, potentially modifiable driver of cost and morbidity.

To this end, effective perioperative blood management in spine surgery must address all phases of care [14,15,16,17]. Preoperatively, this includes optimizing hemoglobin levels, managing anticoagulants, controlling blood pressure, and correcting reversible coagulopathies [5,9,14]. Intraoperative techniques such as administration of tranexamic acid (TXA), use of bipolar cautery, cell salvage, and minimally invasive approaches can help limit visible blood loss. Postoperatively, judicious timing of anticoagulation, drain management, and close monitoring for hematoma formation remain critical [15,16,17]. Despite these strategies, a subset of patients, particularly those with elevated ECI, remain at high risk for disruptive bleeding and related complications. To this end, as the spine surgery population continues to age and become more medically complex, our findings support the need for enhanced preoperative risk stratification and targeted bleeding prevention strategies [9,12,13]. Future studies should aim to refine predictive models and evaluate interventions tailored to high-risk patients, in order to improve both outcomes and value-based care in spine surgery.

This study has several potential limitations. First, it is a retrospective database analysis utilizing administrative claims data, which inherently relies on the accuracy of diagnostic and procedural coding. This introduces the risk of misclassification bias, particularly in identifying and grading the severity of perioperative bleeding events, as well as documenting preoperative anemia and intra- or postoperative hemoglobin trends. Additionally, the analysis did not control for intraoperative factors known to affect bleeding risk in spine surgery, such as the surgical level and approach (anterior versus posterior), use of tranexamic acid, operative time, estimated blood loss, or anesthesia technique. Moreover, the dataset lacked information on long-term clinical outcomes, including patient-reported measures of function, pain, and quality of life, which limits our ability to assess the broader implications of disruptive bleeding beyond the index hospitalization and 90-day postoperative period. Future prospective studies incorporating laboratory or biomarker data would be required to directly correlate molecular mechanisms with clinical bleeding outcomes. Finally, the study cohort was limited to hospitals contributing to the Premier Healthcare Database, potentially underrepresenting certain care settings such as smaller rural facilities or federally funded hospitals, thereby impacting the generalizability of our findings. Despite these limitations, this study represents one of the largest national assessments of bleeding risk in common spine procedures and offers important insights into the influence of comorbidity burden on both clinical and economic outcomes.

## 5. Conclusions

In this study, approximately 9% of patients undergoing spine surgery experienced disruptive bleeding. Importantly, patients with higher comorbidity burdens—as measured by the Elixhauser Comorbidity Index—were at significantly greater risk, reinforcing the need for comprehensive preoperative evaluation and individualized risk assessment. Disruptive bleeding was also strongly associated with increased healthcare utilization, including longer hospital stays, greater use of mechanical ventilation, elevated 90-day readmission rates, and higher inpatient costs. These findings underscore the substantial clinical and economic impact of bleeding complications in spine surgery and highlight the importance of optimizing perioperative blood management strategies to improve outcomes in this medically complex patient population.

## Figures and Tables

**Table 1:** Patient demographics and hospital/provider characteristics compared between patients who had disruptive bleeding versus did not have disruptive bleeding.

Selected Patient Characteristics	Overall (N = 165,461 [100%])	No Disruptive Bleeding (N = 150,124 [90.7%])	Disruptive Bleeding (N = 15,337 [9.3%])
Surgery Type			
Spine Discectomy	8152 (4.9)	7511 (5.0)	641 (4.2)
Spine Fusion	112,421 (67.9)	99,104 (66.0)	13,317 (86.8)
Spine Laminectomy	44,888 (27.1)	43,509 (29.0)	1379 (9.0)
Patient Characteristics			
Age, mean (SD)	59(14)	59(14)	62(13)
Age Category, N (%) a			
18–34	8604 (5.2)	8058 (5.4)	546 (3.6)
35–44	16,796 (10.2)	15,821 (10.5)	975 (6.4)
45–54	30,193 (18.2)	27,906 (18.6)	2287 (14.9)
55–64	43,933 (26.6)	39,735 (26.5)	4198 (27.4)
65–74	43,913 (26.5)	39,053 (26.0)	4860 (31.7)
75+	22,022 (13.3)	19,551 (13.0)	2471 (16.1)
Sex, N (%)			
Women	81,431 (49.2)	73,513 (49.0)	7918 (51.6)
Men	84,030 (50.8)	76,611 (51.0)	7419 (48.4)
Race, N (%)			
African American	14,531 (8.8)	12,790 (8.5)	1741 (11.4)
Asian	1895 (1.1)	1720 (1.1)	175 (1.1)
Other	10,928 (6.6)	9962 (6.6)	966 (6.3)
Unknown	2993 (1.8)	2728 (1.8)	265 (1.7)
White	135,114 (81.7)	122,924 (81.9)	12,190 (79.5)
Hispanic Indicator, N (%)			
Unknown	28,470 (17.2)	26,587 (17.7)	1883 (12.3)
No	128,958 (77.9)	116,312 (77.5)	12,646 (82.5)
Yes	8033 (4.9)	7225 (4.8)	808 (5.3)
Marital Status, N (%)			
Other	13,542 (8.2)	12,121 (8.1)	1421 (9.3)
Unknown	738 (0.4)	679 (0.5)	59 (0.4)
Married	94,338 (57.0)	86,021 (57.3)	8317 (54.2)
Single	56,843 (34.4)	51,303 (34.2)	5540 (36.1)
Payer, N (%)			
Other	16,044 (9.7)	14,686 (9.8)	1358 (8.9)
Commercial	63,408 (38.3)	58,665 (39.1)	4743 (30.9)
Medicaid	13,291 (8.0)	12,140 (8.1)	1151 (7.5)
Medicare	72,718 (43.9)	64,633 (43.1)	8085 (52.7)
Setting of Care			
Inpatient	98,213 (59.4)	85,587 (57.0)	12,626 (82.3)
Outpatient	67,248 (40.6)	64,537 (43.0)	2711 (17.7)
Provider Characteristics			
Hospital Size (No. of beds), N (%)			
500+	60,167 (36.4)	54,194 (36.1)	5973 (38.9)
000–099	7074 (4.3)	6621 (4.4)	453 (3.0)
100–199	19,832 (12.0)	18,185 (12.1)	1647 (10.7)
200–299	26,399 (16.0)	24,054 (16.0)	2345 (15.3)
300–399	27,428 (16.6)	24,813 (16.5)	2615 (17.1)
400–499	24,561 (14.8)	22,257 (14.8)	2304 (15.0)
Teaching Status			
No	78,627 (47.5)	71,186 (47.4)	7441 (48.5)
Yes	86,834 (52.5)	78,938 (52.6)	7896 (51.5)
Urban or Rural, N (%) a			
Rural	13,175 (8.0)	12,076 (8.0)	1099 (7.2)
Urban	152,286 (92.0)	138,048 (92.0)	14,238 (92.8)
Hospital costing type, N (%) aa			
Procedural	117,928 (71.3)	107,251 (71.4)	10,677 (69.6)
RCC	47,533 (28.7)	42,873 (28.6)	4660 (30.4)
Provider Volume, N (%) a			
0–100	19,764 (11.9)	18,537 (12.3)	1227 (8.0)
101–200	25,967 (15.7)	23,762 (15.8)	2205 (14.4)
201–300	24,574 (14.9)	22,126 (14.7)	2448 (16.0)
301–400	20,744 (12.5)	19,091 (12.7)	1653 (10.8)
401–500	16,479 (10.0)	15,118 (10.1)	1361 (8.9)
500+	57,933 (35.0)	51,490 (34.3)	6443 (42.0)
Provider Region, N (%) a			
Midwest	39,503 (23.9)	36,799 (24.5)	2704 (17.6)
Northeast	26,438 (16.0)	23,989 (16.0)	2449 (16.0)
South	81,423 (49.2)	72,445 (48.3)	8978 (58.5)
West	18,097 (10.9)	16,891 (11.3)	1206 (7.9)

Abbreviations: N, number; SD, standard deviation; RCC, ratio of costs-to-charges; %, percentage.

**Table 2:** Patient Distribution by Elixhauser score and presence of disruptive bleeding.

Elixhauser Score	Overall (N = 165,461 [100%])		No Disruptive Bleeding (N = 150,124 [90.7%])		Disruptive Bleeding (N = 15,337 [9.3%])	
0	36,881	22.3	34,707	23.1	2174	14.2
1	41,000	24.8	37,634	25.1	3366	21.9
2	37,475	22.6	33,949	22.6	3526	23.0
3	25,345	15.3	22,695	15.1	2650	17.3
4	13,453	8.1	11,746	7.8	1707	11.1
5	6469	3.9	5520	3.7	949	6.2
6+	4838	2.9	3873	2.6	965	6.3

## Data Availability

The data analyzed in this study are derived from the Premier Healthcare Database (Premier Inc.) and are not publicly available due to licensing and data use agreements. Access to the data may be obtained from Premier Inc. upon reasonable request and with appropriate institutional approvals.
